# Quantitative Guidance for Stove Usage and Performance to Achieve Health and Environmental Targets

**DOI:** 10.1289/ehp.1408681

**Published:** 2015-03-27

**Authors:** Michael A. Johnson, Ranyee A. Chiang

**Affiliations:** 1 Berkeley Air Monitoring Group, Berkeley, California, USA; 2 Global Alliance for Clean Cookstoves, Washington, DC, USA

## Abstract

**Background:**

Displacing the use of polluting and inefficient cookstoves in developing countries is necessary to achieve the potential health and environmental benefits sought through clean cooking solutions. Yet little quantitative context has been provided on how much displacement of traditional technologies is needed to achieve targets for household air pollutant concentrations or fuel savings.

**Objectives:**

This paper provides instructive guidance on the usage of cooking technologies required to achieve health and environmental improvements.

**Methods:**

We evaluated different scenarios of displacement of traditional stoves with use of higher performing technologies. The air quality and fuel consumption impacts were estimated for these scenarios using a single-zone box model of indoor air quality and ratios of thermal efficiency.

**Results:**

Stove performance and usage should be considered together, as lower performing stoves can result in similar or greater benefits than a higher performing stove if the lower performing stove has considerably higher displacement of the baseline stove. Based on the indoor air quality model, there are multiple performance–usage scenarios for achieving modest indoor air quality improvements. To meet World Health Organization guidance levels, however, three-stone fire and basic charcoal stove usage must be nearly eliminated to achieve the particulate matter target (< 1–3 hr/week), and substantially limited to meet the carbon monoxide guideline (< 7–9 hr/week).

**Conclusions:**

Moderate health gains may be achieved with various performance–usage scenarios. The greatest benefits are estimated to be achieved by near-complete displacement of traditional stoves with clean technologies, emphasizing the need to shift in the long term to near exclusive use of clean fuels and stoves. The performance–usage scenarios are also provided as a tool to guide technology selection and prioritize behavior change opportunities to maximize impact.

**Citation:**

Johnson MA, Chiang RA. 2015. Quantitative guidance for stove usage and performance to achieve health and environmental targets. Environ Health Perspect 123:820–826; http://dx.doi.org/10.1289/ehp.1408681

## Introduction

Cookstove programs and enterprises seek to achieve full adoption of high-performing technologies for the nearly 3 billion people who rely on solid biomass fuels to meet their primary household energy demands (Bonjour et al. 2013). Impacts from this solid fuel use include an estimated 4 million premature deaths per year from exposure to health-damaging pollutants (Lim et al. 2012) and substantial climate forcing from the estimated 25% of global black carbon emissions (Bond et al. 2013). Use of inefficient stoves also results in substantial time and monetary burdens from purchasing and collecting fuel (Clancy et al. 2012; García-Frapolli et al. 2010).

Improving emissions and efficiency of cookstoves to address these impacts has long been a focus of stove designers and programs, with a variety of promising new technologies and fuels demonstrating relatively strong performance (Jetter et al. 2012). Efforts to improve cookstove emissions and fuel efficiency have been aided by recent developments in performance standards and guidelines, including the International Organization for Standardization (ISO) International Workshop Agreement *IWA 11:2012, Guidelines for Evaluating Cookstove Performance* (IWA 11:2012; ISO 2012). IWA 11:2012 was agreed upon by a broad, international array of household energy experts and stakeholders, and provides quantitative guidance on *a*) fuel efficiency, *b*) total emissions, *c*) indoor emissions, and *d*) safety. For each of these indicators, IWA 11:2012 outlines “tiers of performance” that specify ranges for product performance based on laboratory testing. The tiers span from performance that is equivalent to traditional three-stone fires (TSF; tier 0), to interim progress (tiers 1–3), and finally to aspirational performance goals (tier 4) (see Supplemental Material, Table S1, for specific tier performance levels for efficiency and indoor emissions tiers). For example, a stove could be measured to be tier 3 for fuel efficiency, tier 3 for total emissions, tier 2 for indoor emissions, and tier 4 for safety. Previous evaluations and comparisons often relied on difficult to define terms like “inefficient,” “clean,” “advanced,” and “improved.” The IWA tiers address the limitations of such terminology and establish quantitative goals for technology developers, as well as help organizations and consumers make informed decisions with technology selection, and drive technology innovation and development (Eichholtz et al. 2010; Lee et al. 2011; Noonan et al. 2012).

Similar guidance has not been provided for cookstove usage, which is also fundamental for attaining health and environmental benefits. Several studies have reported that stove stacking, the use of multiple stoves to meet daily energy demands, is common and the exclusive use of new stove technologies in homes has been rare (Lewis and Pattanayak 2012; Pine et al. 2011; Puzzolo et al. 2013; Ruiz-Mercado et al. 2011). Although it is well understood that continued use of traditional, polluting technologies in homes alongside cleaner stoves and fuels limits potential health and environmental benefits, the extent of traditional stove displacement required to meet air quality and fuel consumption targets is not clear.

To address this need, here we present an approach that extends the “tiers of performance” framework in IWA 11:2012 to provide quantitative guidance that integrates performance and use. Air quality and fuel consumption impacts are estimated for different usage scenarios across ranges of stove performance. The resulting performance–usage scenarios are provided as a tool to help stove designers, program implementers, policy makers, and other stakeholders consider the most appropriate technology and behavior change pathways for achieving maximal impact.

## Methods

Indoor concentrations of PM_2.5_ (particulate matter ≤ 2.5 μm in aerodynamic diameter) and CO (carbon monoxide) were estimated using the single-zone model used for IWA 11:2012 (ISO 2012). Single-zone models have been applied many times for household air pollution studies (Johnson et al. 2011; Prasad et al. 1985; Smith et al. 1983) and are commonly used in air quality and climate research (Apple et al. 2010; Bond et al. 2011; Hellweg et al. 2009). The model predicts concentrations in the kitchen based on emission sources, air exchange rate, and room volume, with the assumption of constant emissions rates and perfect mixing. The model can be described mathematically as

*C_t_* = [*G*/(α*V*)] (1 – *e*^–α^*^t^*) + *C*_o_(*e*^–α^*^t^*), [1]

where

*C_t_* = concentration of pollutant at time *t* (milligrams per cubic meter)
  - = emission rate (milligrams per minute)
α = first order loss rate (nominal air exchange rate) (air exchanges per minute)
  - = kitchen volume (cubic meters)
    - = time (minutes)
*C*_o_ = concentration from preceding time unit (milligrams per cubic meter).
Fuel savings were calculated from the ratios of thermal efficiency as follows:

Percent fuel savings = [1 – (η_T_/η_x_)](percent displacement of the traditional stove), [2]

where η_T_ is the traditional stove thermal efficiency and η_x_ is the new stove thermal efficiency.

The air quality model and fuel savings calculations were applied with the emissions rates and fuel efficiencies shown in Table 1. The emissions rates and thermal efficiencies for the TSF were assumed as IWA 11:2012 tier 0 (for indoor emissions and efficiency), which are based on the TSF’s performance during standardized laboratory tests (Johnson et al. 2012). Tier 4 thresholds for indoor emissions were derived by modeling the stove emission rates required to achieve the World Health Organization (WHO) annual interim 1 target for PM_2.5_ (WHO 2006) and the WHO 24-hr guideline for CO (there is no annual guideline) (WHO 2010). To serve as a reference point for charcoal stoves, the assumed emission rates and thermal efficiencies for a traditional charcoal stove were derived by averaging the four traditional charcoal stoves presented in Jetter et al. (2012). The resulting rounded emission rates were 15 and 1,300 mg/min for PM_2.5_ and CO, respectively, and thermal efficiency was 25%. Traditional charcoal stoves were the Gyapa, ceramic jiko, metal jiko, and Kenya ceramic jiko (Jetter et al. 2012). Emission rates and thermal efficiencies used to represent tiers 1–4 in the model are equidistant between tier boundaries (zero for the lower boundary for indoor emissions tier 4). Thermal efficiency for tier 4 was assumed as 50% by extrapolating from tiers 1–3.

**Table 1:** Emission rates and thermal efficiencies used for modeling air quality and calculating fuel savings, based on IWA 11:2012 tier boundaries.

Stove tier	PM_2.5_ indoor emissions rate (mg/min)		CO indoor emissions rate (mg/min)		Thermal efficiency (%)	
	Value used for model	Range of values for tier	Value used for model	Range of values for tier	Value used for calculation	Range of values for tier
0	40.0	> 40	970	> 970	15	< 15
1	28.5	17–40	795	620–970	20	15–25
2	12.5	8–17	555	490–690	30	25–35
3	5.0	2–8	455	420–490	40	35–45
4	1.0	≤ 2	210	≤ 420	50	≥ 45
Thermal efficiency is based on the high-power phase of the WBT version 4 (WBT Technical Committee 2014).						

Stove usage was incorporated into the model by adjusting cooking times for the respective stoves. A full day of cooking was assumed to be three 1-hr events, as was assumed in IWA 11:2012, and apportioned between the traditional and new stove, ranging from 0% to 100% displacement of the traditional stove with the new stove. Ventilation rates and kitchen volume were kept constant for all model runs, and values were consistent with IWA 11:2012 at 15 air exchanges per hour and 30 m^3^, respectively. The assumptions for cooking time, ventilation rate, and kitchen volume were based on a review of published sources (Bhangar 2006; Cowlin 2005; Johnson et al. 2011; Park and Lee 2003; Raiyani et al. 1993; Smith et al. 1983). To illustrate a typical simulation for predicting daily PM_2.5_ concentrations with 100% TSF usage, the model was run with the aforementioned ventilation rates and kitchen volumes (α and *V*, respectively, in Equation 1), and the tier 0 PM_2.5_ emission rate (*G*) from Table 1 was applied for three distinct 60-min periods to produce minute-by-minute estimates of PM_2.5_ concentrations (*C_t_*).

## Results

**Figure 1:**
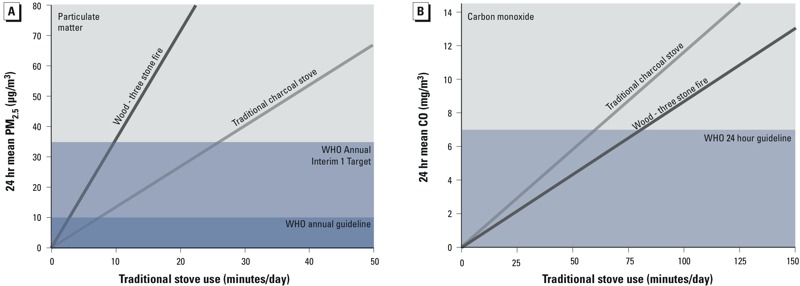
The impact of traditional stove use on concentrations of PM_2.5_ (*A*) and CO (*B*) in the kitchen as a function of three-stone fire and traditional charcoal stove use, as estimated with a single zone air quality model. WHO PM_2.5_ interim 1 target from WHO (2006), and 24‑hr CO guideline from WHO (2010).

*Air quality and traditional stove usage.* Estimates for use of a single stove, assuming linear relationships between stove use and indoor PM_2.5_ and CO, suggest that daily mean concentrations of PM_2.5_ and CO increase rapidly with increased time using traditional stoves (Figure 1). Based on the model, if a TSF (tier 0 for indoor emissions) is used more than approximately 10 min/day (equivalent to 1 hr/week), daily mean concentrations will exceed the WHO interim 1 target for PM_2.5_ of 35 μg/m^3^ (WHO 2006) (Figure 1A), but traditional charcoal stoves could be used for up to approximately 25 min/day (Figure 1A). For the final PM_2.5_ guideline (10 μg/m^3^) (WHO 2006), even 5 min of TSF use per day is estimated to result in exceeding the guideline. The modeled estimates suggest that the TSF can be used for more time before exceeding WHO targets for CO (7 mg/m^3^), with the TSF and charcoal stoves able to be used up to 75 and 50 min/day, respectively, before the 24-hr guideline is surpassed. Stoves that are indoor emissions tiers 1, 2, 3, and 4 are estimated to be able to be used for approximately 15, 30, 75, and 375 min/day before exceeding the WHO guideline for CO (WHO 2006), assuming no other stoves are used.

**Figure 2:**
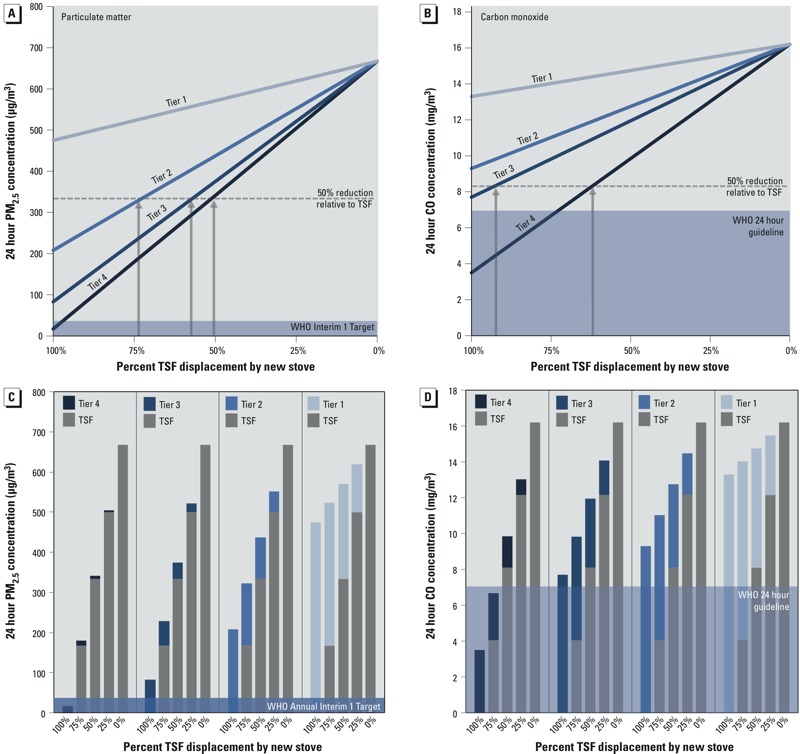
The impact of multiple stove use on air pollutant concentrations in the kitchen as estimated with a single-zone air quality model. Modeled 24-hr mean PM_2.5_ (*A,C*) and CO (*B,D*) concentrations across a range of three-stone fire (TSF) displacement scenarios, which include TSF usage combined with stoves representing indoor emissions tier 1, 2, 3, and 4. Linear relationships between TSF displacement with a new stove and indoor concentrations for PM_2.5_ (*A*) and CO (*B*). Specific contributions from the TSF and indoor emissions tier 1, 2, 3, and 4 stoves to 24-hr PM_2.5_ (*C*) and CO (*D*) concentrations under the different performance–usage scenarios. WHO PM_2.5_ interim 1 target from WHO (2006) and WHO CO 24-hr guideline from WHO (2010).

*Air quality for new stove usage and displacement of the TSF.* The relationships in Figure 1 account only for the contributions of the traditional stove to indoor air quality. When new stoves are introduced into a household, the indoor air quality depends on the emissions contributions from all the stoves being used. When 24-hr mean PM_2.5_ and CO concentrations were modeled across a range of TSF displacement scenarios, including combinations with stoves representing indoor emissions tiers 1, 2, 3, and 4, the only scenario in which WHO targets were reached for PM_2.5_ (Figure 2A) and CO (Figure 2B) were with near complete displacement of the TSF with an indoor emissions tier 4 stove. For PM_2.5_, reaching the WHO interim 1 target of 35 μg/m^3^ (WHO 2006) represents an estimated 92% reduction in kitchen concentrations relative to the assumed baselines scenario with TSFs. However, more modest improvements in indoor air quality can be achieved through multiple performance–usage scenarios. For example, we estimated that a reduction of 50% in 24-hr mean PM_2.5_ concentrations relative to exclusive TSF use could be achieved by indoor emissions tier 2, 3, and 4 stoves displacing approximately 75%, 55%, and 50% of TSF usage, respectively (Figure 2A). Fifty percent relative reductions for CO concentrations compared with exclusive TSF use are estimated to be possible with approximately 90% and 60% displacement of the TSF with indoor emissions tier 3 and 4 stoves, respectively. Supplemental Material, Figure S1, shows the estimated impact on air quality for displacement of traditional charcoal stoves. In addition, because tier levels are bound by upper and lower performance limits, we have also estimated the range of indoor PM_2.5_ and CO concentrations within each respective tier for the different displacement scenarios, which can be found in Supplemental Material, Figure S2.

Under the different performance–usage scenarios, and again assuming linear relationships between stove use and indoor pollutant concentrations, the TSF is estimated to be the dominant source of air pollution for most scenarios. When used for half of the total cooking time, the TSF contributes an estimated 98% of the PM_2.5_ concentrations compared with 2% from the indoor emissions tier 4 stove (Figure 2C). For the same level of displacement with the indoor emissions tier 3 stove, the TSF contributes 89% of the mean 24-hr PM_2.5_ concentrations. For CO, we estimated that the TSF contributes 82% and 68% of indoor concentrations for indoor emissions tier 4 and 3 stove scenarios, respectively, when used for 50% of the cooking time (Figure 2D). The disproportionate air pollutant contributions in relation to stove usage are due to the exponential spacing of the IWA 11:2012 indoor emissions tiers used in our model, which reflect the nonlinear exposure–response relationships of PM_2.5_ with health outcomes such as acute lower respiratory infections (ALRI) (Burnett et al. 2014; Smith et al. 2011). The large estimated contributions from the TSF to indoor pollutant concentrations again underscore the importance of severely limiting their usage to achieve WHO targets.

**Figure 3:**
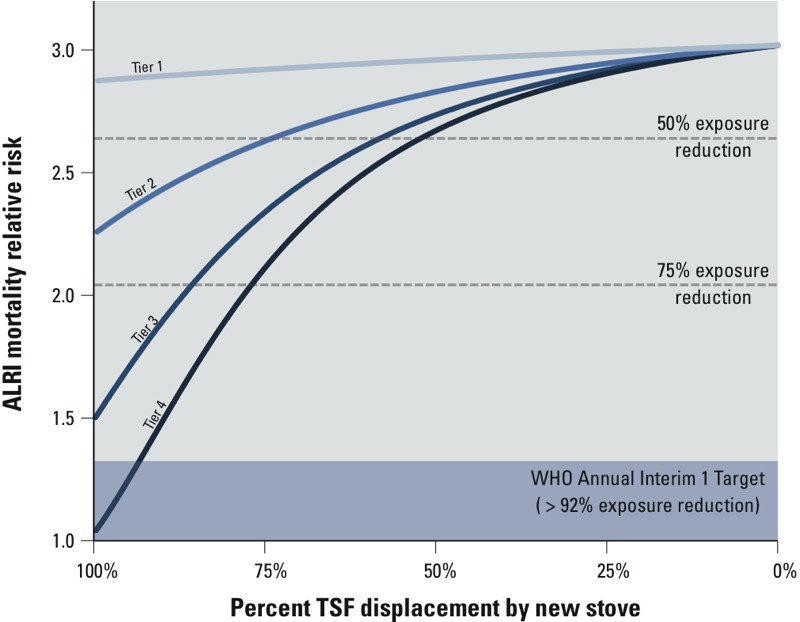
The modeled relative risk of children’s ALRI mortality across various stove performance–usage scenarios, estimated by combining predicted exposures with an exposure–response curve. The gray dashed lines represent exposure reductions of 50 and 75%. Abbreviations: ALRI, acute lower respiratory infection; TSF, three-stone fire. WHO PM_2.5_ interim 1 target from WHO (2006).

In contrast with models that assume linear relationships between stove use and indoor concentrations, PM_2.5_ exposure–response curves for health impacts, such as cardiovascular disease and ALRI, are exponential (Baumgartner et al. 2012; Burnett et al. 2014), which is why the greatest health benefits are accrued by achieving low exposures levels under WHO targets. Reaching these exposure levels is critical, but it is also important to recognize that more modest health gains can be achieved with various technologies and usage scenarios such as those observed for the RESPIRE study (Ruiz-Mercado et al. 2011; Smith et al. 2011). By applying the integrated exposure–risk relationship for household air pollution from the Global Burden of Disease Study 2010 [Institute for Health Metrics and Evaluation (IHME) 2013] and the kitchen/child exposure ratio (0.628) from Smith et al. (2014) to the kitchen concentrations derived from our model (Figure 2A), we estimated that ALRI relative risk for children < 5 years of age could be reduced from approximately 3 to 2 (corresponding to 75% exposure reduction relative to exclusive TSF usage) with indoor emissions tier 3 and 4 stoves displacing 86% and 77% TSF usage, respectively (Figure 3). Twelve percent lower relative risk (corresponding to 50% exposure reduction) could be achieved by displacing a TSF by 73%, 57%, and 51% with indoor emissions tier 2, 3, and 4 stoves, respectively. Reaching the WHO interim 1 PM_2.5_ target of 35 μg/m^3^ (92% exposure reduction) could be achieved with an indoor emissions tier 4 stove displacing 94% of the TSF use, but would still imply a degree of relative risk because the reference level used as a counterfactual to derive the exposure–response curve was 7 μg/m^3^ (IHME 2013). Even with a tier 4 stove achieving 100% displacement, the modeled daily exposure would be approximately 11 μg/m^3^, implying a marginal relative risk (1.03) compared with the counterfactual. Aside from indoor emissions tier 1 stoves, which show no substantive impacts on ALRI relative risk regardless of usage scenario, the modeled estimates indicate that meaningful impacts on ALRI can be achieved for various scenarios of emissions performance and usage. ALRI was used here as the relevant health end point because it is the greatest contributor to the health burden (measured as disability-adjusted life years) associated with household air pollution (Smith et al. 2014), and the exposure–response curve was supported with household air pollution–specific data (Burnett et al. 2014; Smith et al. 2014), although similar relationships with TSF displacement could also be estimated for cardiovascular disease, lung cancer, and other health impacts.

**Figure 4:**
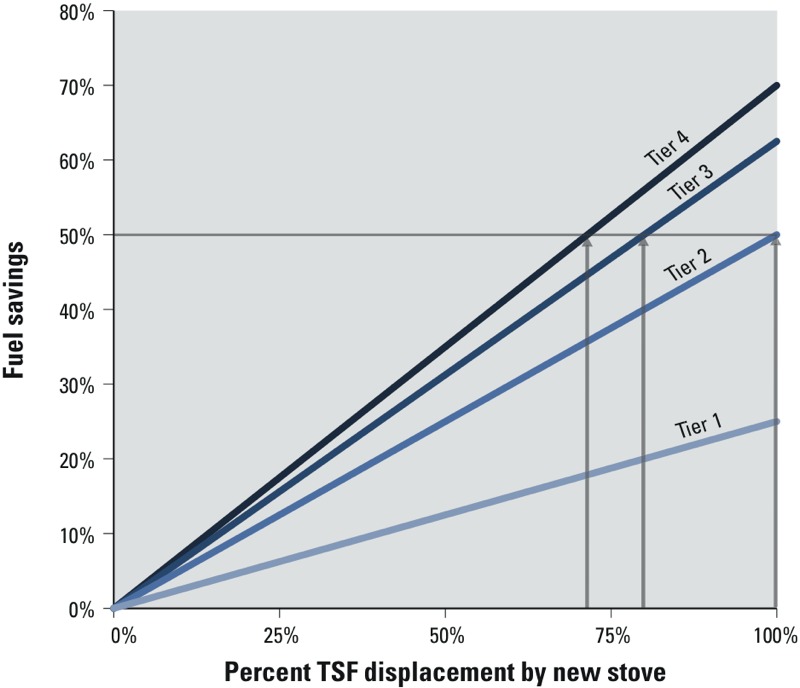
Modeled relationships between three-stone fire (TSF) displacement and fuel savings for different performance–usage scenarios, estimated by the ratio of thermal efficiencies of the new stoves to traditional stoves and the percent displacement of the traditional stove. Gray arrows indicate different performance–usage scenarios for which 50% fuel savings could be achieved.

*Fuel savings and stove usage.* Fuel savings were estimated using Equation 2 and the thermal efficiencies in Table 1, with the resulting linear relationships between usage and fuel savings shown in Figure 4. The highest potential savings of 70% are estimated with thermal efficiency tier 4 stoves completely displacing the TSF. The greatest fuel saving scenarios, although clearly desirable, may not be realistic in many situations where exclusively transitioning to a high-performing stove is difficult. A target of 50% fuel savings, however, is estimated to be achievable by displacing the TSF entirely with a thermal efficiency tier 2 stove, by approximately 80% with a tier 3 stove, or by approximately 70% with a tier 4 stove. Supplemental Material, Figure S3, provides ranges of fuel savings relative to TSFs and traditional charcoal stoves for each thermal efficiency tier level, bounded by upper and lower performance limits.

## Discussion

*Implications for strengthening the clean-cooking sector.* The health and environmental benefits associated with the adoption of a new stove are a function of a cooking system. In addition to stoves and fuels, the cooking system includes user behavior, physical characteristics of the home, cooking practices, and other factors. Each component of the cooking system can be influenced or altered to increase health and environmental benefits. Although the performance–usage model does not account for all of these system components, it integrates many of the quantifiable factors—emissions rates, fuel efficiency, usage and displacement, room size, and ventilation—to illustrate how key parameters influence indoor pollutant concentrations and to explore multiple pathways to reduce household air pollution and fuel use.

**Figure 5:**
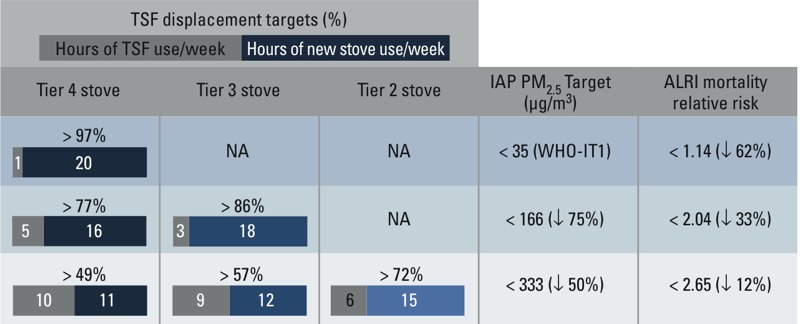
Performance–usage scenarios and associated indoor air pollution (IAP) targets and reductions in ALRI mortality. Given that the percent of three-stone fire (TSF) displacement targets are achieved, the model predicts that it is possible to reach the associated indoor air pollution target and reduction in ALRI mortality. For example, to reach indoor air pollution levels < 166 μg/m^3^, a tier 4 indoor emissions stove would need to be used at least 77% of the time (corresponding to 5 hr using a TSF and 16 hr using a tier 4 indoor emissions stove). The same level of indoor air pollution can be also be reached with a tier 3 indoor emissions stove used at least 86% of the time.

A set of these pathways, based on various performance–usage scenarios, is provided to help organizations make informed decisions on the interventions most likely to achieve their respective goals (Figure 5). The same indoor air pollution targets and reductions in ALRI relative risk can be achieved with different combinations of displacement and stove emissions performance.

In cases where full adoption of a high-performing stove is difficult to achieve, the framework presented here can help programs and enterprises evaluate appropriate combinations of performance and usage. The longer term goals are to concurrently maximize new stove performance, adoption of new stoves, and displacement of old stoves. Opportunities to achieve these goals, including for program implementers, stove designers, and distributors, are discussed below.

*Translating health and fuel use goals into implementation.* While meeting WHO air quality guidance is the surest way to protect health, we estimate that more modest targets, such as reducing kitchen concentrations of PM_2.5_ to < 166 μg/m^3^ or < 333 μg/m^3^, which may be achieved through multiple performance–usage pathways, would reduce the relative risk of ALRI mortality by 33 and 12%, respectively, compared with the TSF-only use scenario (Figure 5). Although a high-performing stove with less displacement could be equivalent to a low-performing stove with more displacement (Figure 2A,B), the largest impacts are realized only with near complete displacement of the TSF with use of low-emissions technologies (Figure 2C,D). These results highlight the enormous emissions contributions of a TSF relative to new stoves. Even minimal use of the TSF quickly raises concentrations to levels above WHO thresholds, where the exposure–response curves begin to level out, making health gains more difficult to achieve. The importance of exclusive or near exclusive use of a new stove is also supported by the RESPIRE study, which showed the impact of a chimney stove on reducing incidence of ALRI (Smith et al. 2011). Indoor air pollution and personal exposures were reduced by 90% and 50%, respectively, but these reductions were aided by weekly field team visits to ensure that the chimney stoves were well maintained and working properly. Thus, efforts to expedite the transition to clean fuels (e.g., liquefied petroleum gas, ethanol) and technologies with the ability to fully displace traditional cookstoves should be the ultimate priority.

As is the case with meeting health goals, the best option for fuel savings is exclusive use of a high-performing stove. Our model-based estimates of fuel savings (Figure 4) may be used to identify the optimal balance of fuel performance and usage for a specific context. Cookstove programs should strongly consider balancing the usability and technical performance of a stove when aiming for specific savings targets. For example, high-performing stoves, in comparison with less-fuel-efficient stoves, can require more fuel preparation, such as drying wood and cutting into small pieces, which may limit the usage of these types of stoves. Stove designs that do not require as much fuel processing while maintaining performance are discussed below. In addition, there are opportunities for fuel-processing enterprises to provide an affordable fuel alternative that would eliminate the need for users to process fuel at the household level.

Behavior-change strategies can also be used to increase the usage of high-performance stoves and displacement of the TSF, or to mitigate the impact of emissions. The application of this quantitative guidance on household energy activities with behavior change components was explored previously (Johnson and Chiang 2015).

*Stove designers: improving performance and usability.* Usage is ultimately determined by consumers and is not typically integrated into standards frameworks. This performance–usage model, however, complements the existing performance targets in IWA 11:2012 with quantitative guidance that designers can use in their development process. TSF displacement targets, for example, can help designers ensure that their high-performing technologies are well suited for the fraction of cooking tasks that correspond to the desired indoor air pollution reduction (Figure 5) and fuel savings targets (Figure 4).

*Distributors and retailers: selecting and marketing products.* Distributors and retailers use information on performance and suitability of stoves to provide products that meet user needs. Ideally, independent evaluations of performance, usage, and consumer preferences are used to help identify products best suited for a given context. These evaluations can be shared through resources such as the Clean Cooking Catalog (http://catalog.cleancookstoves.org), a global database of stoves and test results designed to provide clarity for evaluating stove options. Information from the catalog on stove characteristics (e.g., compatibility with different pot types) can be evaluated along with performance and user preferences to determine which technologies are likely to result in the best performance–usage scenarios.

Marketing messages about new technologies often attribute the benefits to the technology alone, rather than to the use of the technology. Any fuel savings or health benefits are achieved only if new stoves are used and replace traditional technologies; thus, this message should be communicated by distributors and retailers who interface with consumers. For product marketing and broader consumer-awareness campaigns, communicating this message can be challenging, especially in cases when consumers do not respond well to negative messages about current products (Pascaud and Thivillon 2014). However, marketing and consumer-awareness campaigns should consider ways to encourage higher levels of use of the new technology and displacement of traditional technologies.

*Measuring stove usage.* Research and monitoring efforts often focus on the new technology or intervention, as well as on factors that influence adoption of new technologies. Understanding how new technologies and interventions perform is a fundamental component to assessing air quality, health, fuel consumption, and other outcomes. The analysis presented here, however, indicates that traditional stove use, even at relatively low usage rates, drives air pollutant concentrations. Thus, research and monitoring efforts should also account for use of traditional technologies and factors that influence their use and displacement.

Explicitly connecting traditional stove use with impacts and program effectiveness requires a means to measure or estimate stove usage. Measuring progress against the usage targets in Figures 4 and 5, for example, require that quantitative stove-use estimates be made. Quantitative stove-use data, such as stove temperature measured over time (Ruiz-Mercado et al. 2013), support investigations into how user behavior, usage patterns, and stove performance are directly related to household air pollution, personal exposure, and fuel consumption impacts.

*Recommendations for future modeling of usage and performance.* Modeling the cooking system. As highlighted above, the system impacting kitchen concentrations and exposures includes a variety of factors and sources that are not fully addressed in the model, such as household lighting, trash burning, and neighborhood pollution, as well as behavioral considerations such as fuel-processing practices and adjustment of ventilation conditions. If other emissions sources or solid fuel use within the community are great enough, the impact of household-level interventions may be limited by high ambient contributions to household air quality. Future modeling that considers multiple households in a community would provide guidance on what level of adoption is needed within a community to reach specific targets for air quality.

Ventilation is particularly important because it substantially affects indoor air quality (Baumgartner et al. 2011; Johnson et al. 2011; Nazaroff 2008). A systematic laboratory study showed that ventilation can reduce indoor concentrations of PM_2.5_ by as much as 60% (Ruth et al. 2013). In rural Rwanda, median indoor PM_2.5_ concentrations were half as much for homes cooking outdoors compared with homes cooking indoors (Rosa et al. 2014). In addition, ventilation can be part of or the primary intervention strategy based on behavioral or physical changes in the household. In a previous study (Johnson and Chiang 2015), we explored the implications of ventilation’s impact on stove usage, finding that in comparison with the IWA 11:2012 ventilation rate of 15 ACH (air changes per hour), higher ventilation rates of 25–45 ACH would allow TSF usage for two to three times longer before WHO PM_2.5_ targets are exceeded. These variations in the cooking system can be addressed through probabilistic modeling such as described previously (Johnson et al. 2011), where a fuller analysis of this cooking system variability was presented by applying a Monte Carlo approach to a similar single-zone model.

There are other important considerations that the framework does not account for, including the availability and renewability of fuel resources. Displacing unsustainable charcoal with renewably sourced pellets, for example, has tremendous ecological and environmental benefits regardless of the efficiency of stoves that use processed fuels (Chidumayo and Gumbo 2013; Ghilardi et al. 2013).

Baseline and stacking scenarios. There are a variety of different baseline stoves and stacking scenarios that vary across regions and demographics. The use of a TSF or traditional charcoal stove as a reference point is not strictly applicable for many contexts. In terms of absolute usage of the traditional stove and its impact on air quality, however, the assumption of a TSF or traditional charcoal stove will provide a relatively conservative estimate of emissions contributions from traditional stoves.

When a new stove is introduced into a household, the total time that cooking devices are used can change or even increase. The model used here to compare scenarios held total cooking time constant at 3 hr, which is a simplification because total cooking time in homes can be higher or lower. Modeling other stacking scenarios in which the introduction of new cooking devices changes total cooking time could provide a more specific guidance for such cases.

Model limitations. The indoor emission rates used in the model are based on controlled laboratory tests, which are known to underestimate emissions relative to normal daily stove use in homes (Chen et al. 2012; Johnson et al. 2010). Higher emission rates would require even lower levels of TSF use to stay within WHO targets, and daily cooking times > 3 hr would imply that new stoves need to be cleaner to result in the same indoor pollutant concentrations modeled here. For example, mean cooking times in India have been estimated to range from 3.1 to 4.6 hr/day (Bhangar 2006; Raiyani et al. 1993; Smith et al. 1983). There are other assumptions, however, in the model that are more conservative. For example, the model assumes that all emissions enter the room and fully mix, whereas in most homes a large fraction of the emissions plume exits through windows, eaves, or other openings and never mixes throughout the kitchen. As a first step toward providing straightforward and practical guidance on stove usage, however, here we have focused on only the IWA 11:2012 scenario.

Future laboratory and field studies of stove performance and usage could also use this framework to develop metrics and collect data that integrates emissions, fuel use, and stove usage. Results from these studies would provide empirical data to strengthen the model, especially when usage measurements are combined with measurements of fuel use, emissions, indoor air pollution, and kitchen parameters, as was done for a case study in India (Johnson et al. 2011). Assessing model performance across a range of usage-performance scenarios in homes would be especially helpful. Perhaps most critical would be understanding how the model performs as lower emission technologies approach near exclusive use, where the predicted indoor air concentrations begin to approach WHO guidance levels because this is where the usage guidance is most relevant. Ideally, refinements of the model to account for location or specific factors such as ventilation rates, cooking times, and others would help provide more applicable guidance for specific contexts.

## Conclusions

The importance of both performance and usage on achieving impacts has long been recognized within the household energy sector. This conclusion is reinforced by performance–usage modeling results. The quantitative framework also provides specific guidance for how performance and usage combine to influence household air pollution, which leads to practical implications for different stakeholders within the sector. Although achieving high levels of both performance and adoption is a tremendous challenge, especially at a global scale, this framework can help the household energy sector prioritize efforts in the short term and achieve continuous improvement over the long term.

## Supplemental Material

(622 KB) PDF
