# Supplementary material for: Quantitative Guidance for Stove Usage and Performance to Achieve Health and Environmental Targets
Source: Environ Health Perspect. 2015 Mar 27;123(8):820–6. doi: 10.1289/ehp.1408681 (PMC4529010; doi:10.1289/ehp.1408681)
Supplement: (622 KB) PDF [file ehp.1408681.s001.acco.pdf]

Environ Health Perspect
doi: 10.1289/ehp.1408681

Note to Readers: ***EHP*** **strives to ensure that all journal content is accessible to all readers. However, some figures and Supplemental Material published in *EHP* articles may not conform to 508 standards due to the complexity of the information being presented. If you need assistance accessing journal content, please contact ehp508@niehs.nih.gov. Our staff will work with you to assess and meet your accessibility needs within 3 working days.**

Supplemental Material

Quantitative Guidance for Stove Usage and Performance to Achieve Health and Environmental Targets

Michael A. Johnson and Ranyee A. Chiang

Supplemental material contains the corresponding figures to those of Figures 2 and 3 in the main text, but with charcoal stoves. Figures 2a, 2b, and 4 of the main text are also presented here with the additions of IWA 11:2012 tier level boundaries.

Table of Contents

Figure S1. The impact of multiple stove use on air pollutant concentrations in the kitchen as estimated with a single zone air quality model. Modeled 24 hour mean $PM_{2.5}$ and CO concentrations across a range of traditional charcoal stove displacement scenarios, which include traditional charcoal usage combined with stoves representing Indoor Emissions Tier 1, 2, 3, and 4. Graphs 2A and 2B show the linear relationships between traditional charcoal stove displacement with a new stove and indoor concentrations for $PM_{2.5}$ (A) and CO (B). Graphs 2C and 2D show the specific contributions from the traditional charcoal stove and Indoor Emissions Tier 1, 2, 3, and 4 stoves to 24 hour $PM_{2.5}$ and CO concentrations under the different performance-usage scenarios.

Figure S2. The impact of multiple stove use on air pollutant concentrations in the kitchen as estimated with a single zone air quality model. Modeled 24 hour mean $PM_{2.5}$ and CO concentrations across a range of three-stone-fire displacement scenarios, which include three-stone-fire usage combined with stoves representing Indoor Emissions Tier 1, 2, 3, and 4. Graphs 2A and 2B show the linear relationships between three-stone-fire displacement with a new stove and indoor concentrations for $PM_{2.5}$ (A) and CO (B). Boundaries of the IWA 11:2012 indoor

emissions tiers have been added to illustrate the range of modeled outcomes within each respective tier.

Figure S3. Modeled relationships between three-stone-fire displacement and fuel savings for different performance-usage scenarios, estimated by the ratio of thermal efficiencies of the new to traditional stoves and the percent displacement of the traditional stove. Boundaries of the IWA 11:2012 fuel efficiency tiers have been added to illustrate the range of outcomes possible within each respective tier.

Table S1: ISO IWA Tiers for Indoor Emissions and Efficiency.

References

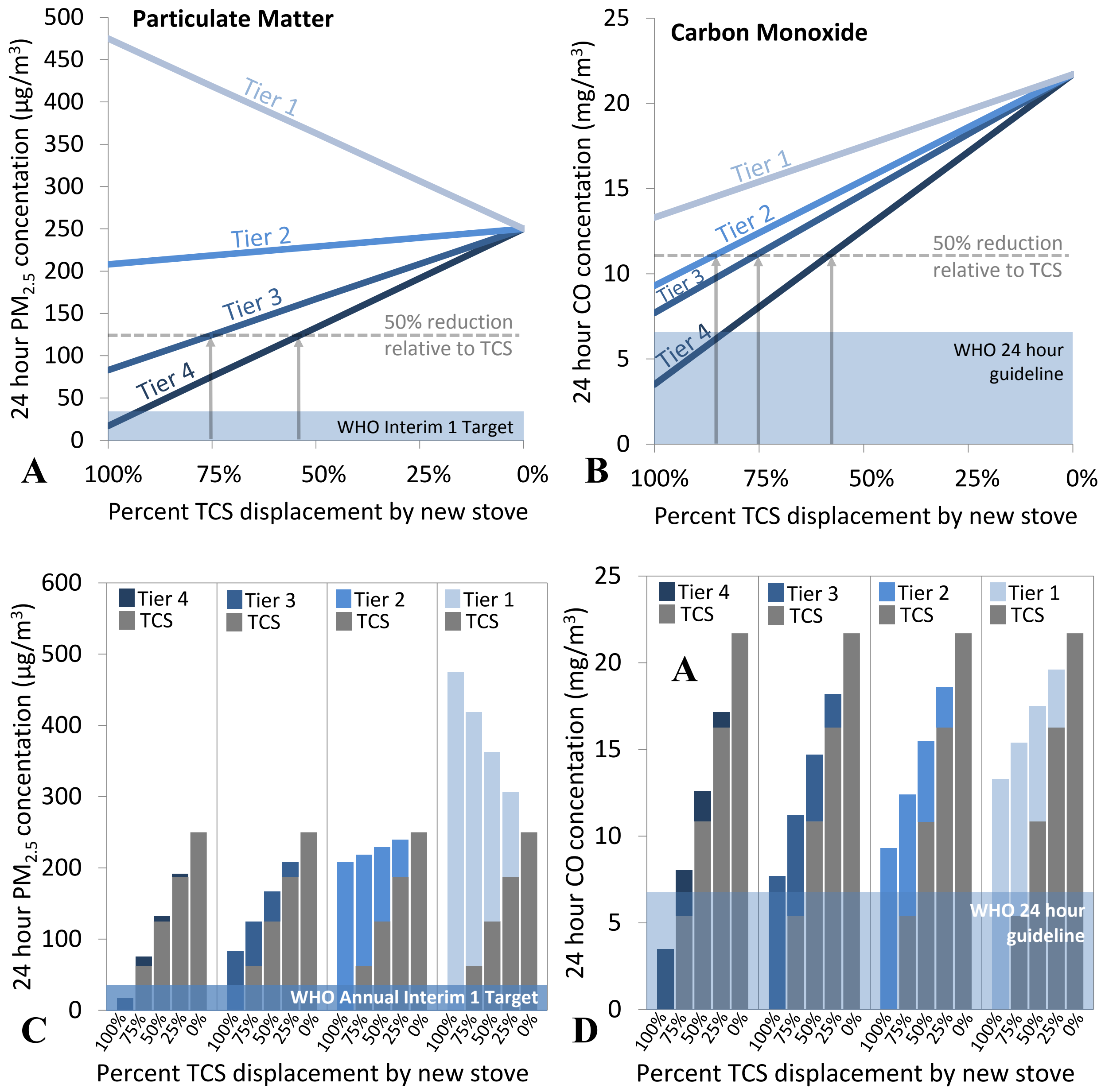

Notes: TCS = traditional charcoal stove; source for WHO Annual Interim 1 Target $PM_{2.5}$ (WHO 2006); source for WHO 24 hour CO guideline (WHO 2010).

Figure S1. The impact of multiple stove use on air pollutant concentrations in the kitchen as estimated with a single zone air quality model. Modeled 24 hour mean $PM_{2.5}$ and CO concentrations across a range of traditional charcoal stove displacement scenarios, which include traditional charcoal usage combined with stoves representing Indoor Emissions Tier 1, 2, 3, and 4. Graphs 2A and 2B show the linear relationships between traditional charcoal stove displacement with a new stove and indoor concentrations for $PM_{2.5}$ (A) and CO (B). Graphs 2C

and 2D show the specific contributions from the traditional charcoal stove and Indoor Emissions Tier 1, 2, 3, and 4 stoves to 24 hour $PM_{2.5}$ and CO concentrations under the different performance-usage scenarios.

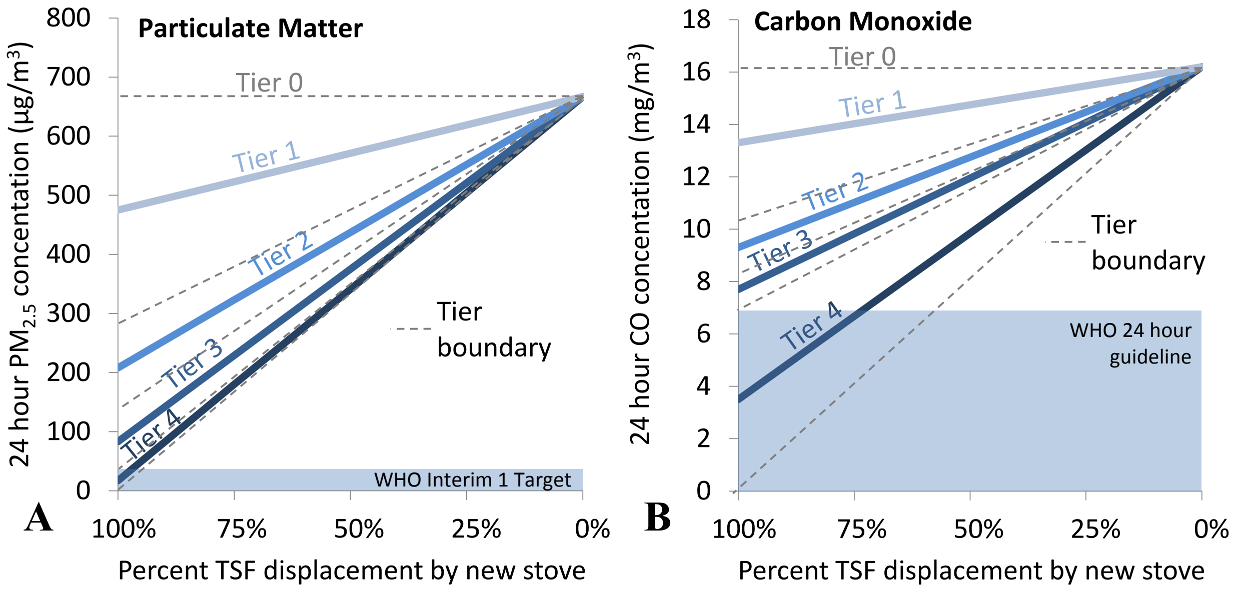

Notes: TSF = three-stone-fire; source for WHO Annual Interim 1 Target $PM_{2.5}$ (WHO 2006), source for WHO 24 hour CO guideline (WHO 2010).

Figure S2. The impact of multiple stove use on air pollutant concentrations in the kitchen as estimated with a single zone air quality model. Modeled 24 hour mean $PM_{2.5}$ and CO concentrations across a range of three-stone-fire displacement scenarios, which include three-stone-fire usage combined with stoves representing Indoor Emissions Tier 1, 2, 3, and 4. Graphs 2A and 2B show the linear relationships between three-stone-fire displacement with a new stove and indoor concentrations for $PM_{2.5}$ (A) and CO (B). Boundaries of the IWA 11:2012 indoor emissions tiers have been added to illustrate the range of modeled outcomes within each respective tier.

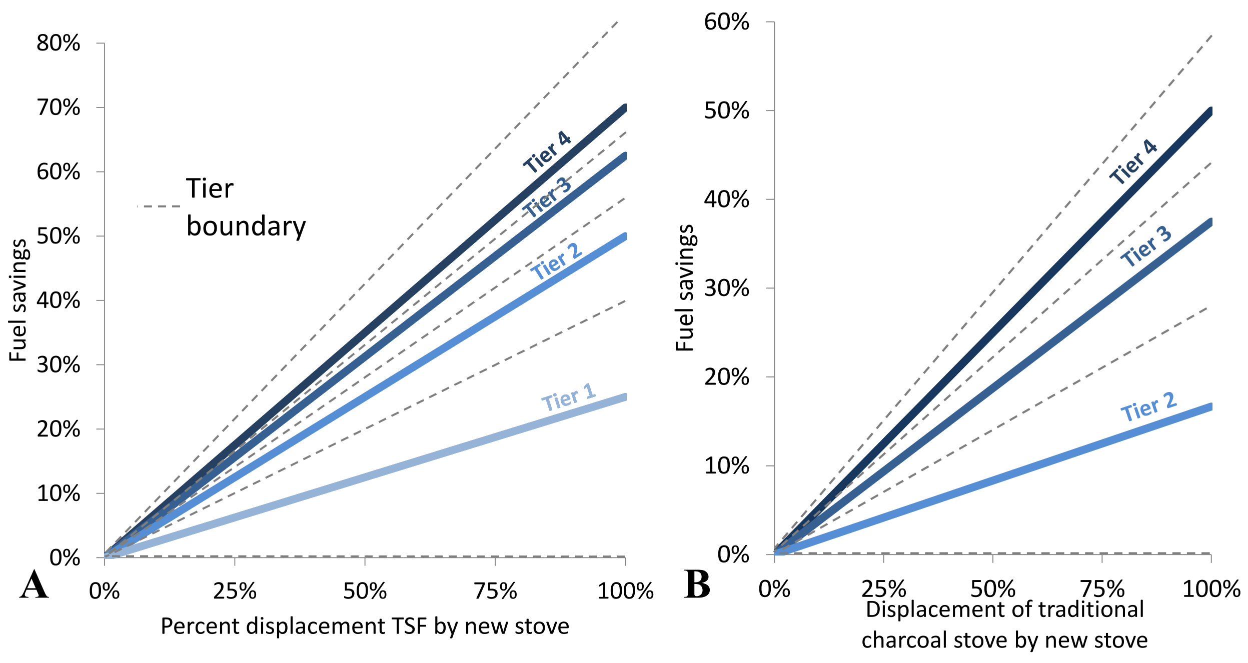

Notes: TSF = three-stone fire. TCS = traditional charcoal stove.

Figure S3. Modeled relationships between three-stone-fire displacement and fuel savings for different performance-usage scenarios, estimated by the ratio of thermal efficiencies of the new to traditional stoves and the percent displacement of the traditional stove. Boundaries of the IWA 11:2012 fuel efficiency tiers have been added to illustrate the range of outcomes possible within each respective tier.

Table S1: ISO IWA Tiers for Indoor Emissions and Efficiency.

**Indoor emissions Sub-tiers**		
	**Indoor emissions CO (g/min)**	**Indoor emissions $PM_{2.5}$ (mg/min)**
**Tier 0**	>0.97	>40
**Tier 1**	≤0.97	≤40
**Tier 2**	≤0.62	≤17
**Tier 3**	≤0.49	≤8
**Tier 4**	≤0.42	≤2

**Efficiency/fuel use Sub-tiers**		
	**High power thermal efficiency (%)**	**Low power specific consumption (MJ/min/L)**
**Tier 0**	<15	>0.050
**Tier 1**	≥15	≤0.050
**Tier 2**	≥25	≤0.039
**Tier 3**	≥35	≤0.028
**Tier 4**	≥45	≤0.017

The overall Tier for Indoor Emissions and Efficiency is the minimum Sub-tier value within that indicator category. For example, the Indoor Emissions Tier is the minimum of the Sub-tiers for $PM_{2.5}$ and CO emissions. Additional Tier values are available at http://cleancookstoves.org/technology-and-fuels/standards/iwa-tiers-of-performance.html. These tiers were aligned with Water Boiling Test 4.2.3 and Biomass Stove Safety Protocol 1.1 (http://cleancookstoves.org/technology-and-fuels/testing/protocols.html).

References

WHO. 2010. *WHO guidelines for indoor air quality: selected pollutants*. World Health Organization Regional Office for Europe, Bonn.

WHO. 2006. *WHO guidelines for particulate matter, ozone, nitrogen dioxide and sulfur dioxide*. World Health Organization Press, Geneva.
